# Relations of the Big-Five personality dimensions to autodestructive behavior in clinical and non-clinical adolescent populations

**DOI:** 10.3325/cmj.2012.53.450

**Published:** 2012-10

**Authors:** Marina Kotrla Topić, Marina Perković Kovačević, Boris Mlačić

**Affiliations:** 1 Ivo Pilar Institute of Social Sciences, Zagreb, Croatia; 2 Clinical hospital Osijek, Osijek, Croatia

## Abstract

**Aim:**

To examine the relationship between the Big-Five personality model and autodestructive behavior symptoms, namely Autodestructiveness and Suicidal Depression in two groups of participants: clinical and non-clinical adolescents.

**Methods:**

Two groups of participants, clinical (adolescents with diagnosis of psychiatric disorder based on clinical impression and according to valid diagnostic criteria, N = 92) and non-clinical (high-school students, N = 87), completed two sets of questionnaires: the Autodestructiveness Scale which provided data on Autodestructiveness and Suicidal Depression, and the International Personality Item Pool (IPIP), which provided data on the Big -Five personality dimensions.

**Results:**

Clinical group showed significantly higher values on the Autodestructiveness scale in general, as well as on Suicidal Depression, Aggressiveness, and Borderline subscales than the non-clinical group. Some of the dimensions of the Big-Five personality model, ie, Emotional Stability, Conscientiousness, and Agreeableness showed significant relationship (hierarchical regression analyses, *P* values for β coefficients from <0.001 to 0.021) with Autodestructivness and Suicidal Depression, even after controlling for the sex and group effects or, when analyzing Suicidal Depression, after controlling the effect of other subscales.

**Conclusion:**

The results indicate that dimensions of the Big-Five model are important when evaluating adolescent psychiatric patients and adolescents from general population at risk of self-destructive behavior.

In the past few years, autodestructive behavior has been acknowledged as a serious disease and cost factor in an increasing number of different medical cases (1). Autodestructive tendencies can manifest in different ways – as acute or chronic, latent or overt, conscious or subconscious (2). In studies on autodestructive behavior, researchers usually focus on intentional, self-directed destruction of body tissue without suicidal intent (1,3-8). Autodestructive behavior is often referred to as “self-destructive behavior,” “self-injurious behavior,” “self-injury,” “self-harm,” “self-mutilation,” or “cutting” (8).

For a long time it was considered that autodestructive behavior was related primarily to emotional disturbances (9,10). Therefore, most of the research on this topic was done using clinical populations. Only in the last decade or so, the research began focusing on other populations, noticing that there are high rates of different kinds of autodestructive behavior among youth and young adults in general population. This finding brought out the importance of other factors relevant for the development of autodestructive behavior, besides emotional disturbance, such as different social and cultural factors (4). It also highlighted some concerns regarding instruments designed for capturing and measuring symptoms of this kind of behavior in clinical and in non-clinical populations.

## Psychopathology and personality assessment

When assessing personality characteristics of psychiatric patients, clinicians frequently use questionnaires aimed at discovering psychopathological symptoms (11), but some researchers argue that many kinds of abnormal behavior, including autodestructive behavior, can be viewed as exaggerated versions of normal personality traits. This would mean that in some patients with diagnosed psychological disorders, psychopathology could be seen as falling on a continuum with normal personality instead of being regarded as a distinct departure from normal (12,13). One of a number of alternative dimensional models of personality disorders (14) and other psychopathological symptoms that has sustained a great deal of attention is the Big-Five personality model (15,16).

## The Big-Five personality model

The emergence of this model is considered as one of the most significant developments in the history of personality psychology (17). There are several types of this model, but the fundamental model was originally associated with studies of personality traits used in natural language. The model has five broad dimensions: Extraversion/Introversion, Agreeableness, Conscientiousness, Emotional Stability/Neuroticism, and Intellect (18). Each of the lexical Big-Five factors consists of nine facets (19).

A number of studies have examined the relationship between the Big-Five personality model and mental health problems in clinical and normal-range populations. Focusing most often on personality disorders in adult populations, their results point to a strong relationship between mental health problems and four domains of the Big-Five model: Neuroticism, Extraversion, Agreeableness, and Conscientiousness (20-22), with less consistent data regarding the role of Intellect. Recently studies have moved their focus to developmental antecedents of personality pathology in adolescence, or even childhood, trying to identify the role of general personality according to the Big-Five model in describing personality disorders and their precursors (16,22-25). Since then, the use of the Big-Five personality model and its questionnaires has been suggested to be a valid adjunct to usual clinical instruments when it comes to diagnosing and planning a treatment. The question that remains to be answered is the nature of the relationship of the Big-Five and other mental health problems, namely autodestructive behavior. The systematic research in this area is scarce, a reason for which might be that the manifestations of autodestructive behavior are often incorporated in a wide area of different mental health problems and not studied separately. For example, autodestructive behavior is often studied in the context of attempted suicide; therefore it is sometimes difficult to discern autodestructive behavior from other mental health problems. The relationship between autodestructive behavior and suicide is a complex one, but it is generally considered that there is a strong correlation between these two phenomena, as well as between autodestructive behavior and depression.

Since autodestructive behavior is strongly related to suicidal ideation and depression, the findings on the relationship between these two phenomena and the Big-Five can give valid guidelines for the research on the Big-Five and autodestructive behavior symptoms. Studies show that suicidal ideation, as one type of autodestructive behavior symptom, is associated with low Emotional Stability, low Extraversion, and low Agreeableness (26). The studies investigating depression, which is often closely related to autodestructive behavior, show that a high level of Neuroticism was significantly associated with recurrence of depression (27). As for the relationship of other factors with depression, research points to a negative association of depression with Extraversion (28,29) and Emotional Stability (29), and a positive association of depression with Openness to Experience (30). Also, depressed individuals score low on Conscientiousness (31).

This study addressed the adolescent population. While there is substantial research that shows age-related changes in some of the Big-Five dimensions, especially in the adolescence (32,33), studies on adolescents’ psychiatric problems and their relations with the Big-Five are rare or non-existing. Furthermore, those rare studies point to inconsistent findings, with some reporting significant negative correlations between depressive symptomatology or similar internalized behavior syndromes and two Big-Five dimensions: Emotional Stability and Extraversion (34), or Emotional Stability and Conscientiousness (35), as well all five dimensions (36).

Our main research question pertained to how well the Big-Five personality model could account for Autodestructive behavior symptoms, namely Autodestructiveness and Suicidal Depression in adolescence.

## Method

### Participants

The sample consisted of two groups, clinical and non-clinical. The clinical group consisted of 92 adolescents (52 female) who were treated in ambulatory care or hospitalized at the Child and Adolescent Psychiatric Ward of the Psychiatric clinic at the Clinical Hospital Osijek, and who agreed to participate in the research. The participants from the clinical group were all given a diagnosis of psychiatric disorder. The diagnosis was based on the clinical impression and according to valid diagnostic criteria (37). Their mean age was 15.77 years (range 13-20). They were treated for different problems diagnosed according to DSM-IV criteria (37): behavioral disorders (38%), anxious disorders (23%), depressive disorders (16%), psychotic disorders (7%), eating disorders (5%), and other disorders (10%). The non-clinical group consisted of 87 adolescents (65 female) from two Osijek high schools. Their mean age was 15.57 years (range 13-18). The high schools were chosen randomly from a list of Osijek high schools, and in each school we randomly selected one class of students, the only important factor being that participants match the clinical group in age. From those two classes, all the students whose parents gave their consent, were included in the research.

### Procedure

The clinical group data were collected by one of the authors during the standard psychological testing procedure at the Child and Adolescent Psychiatric Ward. The approval for the data collection was given by the participants’ parents who accompanied them to the treatment, and participants themselves were explained the purpose of the research. The non-clinical group data were collected during school hours by the authors. Parents of the non-clinical participants gave their consent prior to the study by signing the letter given to them by their child. The administration of the questionnaires for both groups lasted about 20 minutes. Ethical approval was received from the Ethics Committee of the Ivo Pilar Institute of Social Sciences, and from the Ethics Committee of the Clinical Hospital Osijek.

### Measures

*Autodestructiveness scale* (2). The scale consists of 107 items for which respondents gave “yes” or “no” answers, depending if the statement could be applied to them or not. Each “yes” answer was later given 1 point, and the final score for the respondent on the scale was the sum of “yes” ratings for all of the items. The internal consistency reliability (coefficient α) for this measure was 0.94, in accordance with previous research that also showed comparable reliability (38). The total result on this scale pointed to the extent of disturbance of the autodestructive drive control (2) and it was referred to as Autodestructiveness.

Previous factor analyses of the items from this scale (2) pointed to four factors, which corresponded to four subscales: Suicidal Depression, Anxiety, Aggressiveness, and Borderline subscale. In previous research, each subscale showed acceptable reliability (38). Besides the total score on Autodestructiveness scale, results on these subscales were often considered for clinical purposes, especially since each of the subscales coincides to specific DSM-IV disorder classification.

The subscale of Suicidal Depression consists of 32 items and measures the feelings of inferiority, hopelessness, lack of tolerance to frustration, lack of self-control, suicidal ideas, and suicide attempts. Anxiety subscale consists of 32 items measuring fear, tendency to worry, feeling of personal adequacy, lack of self-esteem, and tendency to avoid activities by over exaggerating potential risks and dangers. Aggressiveness subscale consists of 22 items measuring psychopathic aggressive behavior, impulsivity, lack of self-control and empathy, tendency to engage in high risk activities, and self-hurt types of behavior. Borderline subscale consists of 21 items measuring disturbances in interpersonal relationships, lack of altruism and empathy, instability of emotional relationships, specific autistic system of thought, and tendency to autodestructive behavior, including suicide attempts.

*International Personality Item Pool (IPIP)* (39). To measure the Big-Five domains we used the Croatian translation (39) of the IPIP Big-Five domains (40), with 50 items (short form). Participants were asked to read each of the 50 items and then rate how well they believed it described them on a 5-point Likert-type scale ranging from 1 (very inaccurate) to 5 (very accurate) as in the original instrument (40). Previous research confirmed the stability of the five-factor structure of IPIP50 and satisfactory scale reliability on a sample of Croatian adolescents (41). The IPIP50 consists of 10 statements for each of the five factors of the Big-Five model: Extraversion, Agreeableness, Conscientiousness, Emotional Stability, and Intellect. High results on Extraversion point to pronounced engagement with other people and with the external world. Extraverts are sociable, outgoing, energetic, lively, and prefer to be around people, while introverts are reserved, sober, and quiet (42). Agreeableness reflects individual differences in peoples’ interest in needs and well-being of others, therefore high agreeableness is marked by altruism, caring, and emotional support (43). Conscientiousness reflects the way in which we control, regulate, and direct our impulses, and high conscientiousness points to a reliable, well organized, responsible, and hard-working person (44). Emotional Stability/Neuroticism is marked by a tendency to get less easily upset and less emotionally reactive. High emotional stability indicates calm, composed, and relaxed individuals who do not react with intense emotions (45). High results on the Intellect dimension point to an imaginative person who enjoys variety, novelty, and change, and who has intellectual and artistic interests.

### Statistical analysis

Data were analyzed using SPSS software for Windows, version 20.0 (2012., IBM Corporation, Software Group, Armonk, NY, USA). The Kolmogorov-Smirnov test showed that all the scales had normal distribution in both groups, clinical and non-clinical. After the reliability analyses of all the variables encompassed by the study, which was done using Cronbach α coefficients in total sample and in both samples individually, we first tested the differences between clinical and the non-clinical group in Autodestructiveness scale and all its subscales, as well as in Big-Five scales, using *t* tests. We then calculated Pearson correlation coefficients for all the variables in the study. To answer the main research questions of the study, we performed a set of three hierarchical regression analyses with Autodestructiveness as the criterion variable (one for the total sample and one for each of the two groups), and other three hierarchical regression analyses with Suicidal Depression as the criterion variable. Hierarchical regression analyses enabled us to determine the order in which variables are entered into the regression equation, allowing us then to examine the contribution of variables entered later, above and beyond the first group of independent variables.

## Results

### Reliability analyses, descriptive statistics, and differences in Mean Scale Scores

A majority of the scales had acceptable to high reliabilities (Table 1), however a few scales in the subsamples fell below the desired value of 0.7 and these results should be interpreted with caution. Those few scales that had internal consistencies lower than reported in the literature could have resulted with lower correlations between the observed constructs. In the case of Autodestructivness (total score) and all of its subscales, Cronbach α coefficients were somewhat lower in the non-clinical than in clinical sample, but still had acceptable values. The Big-Five scales had somewhat higher reliabilities in the non-clinical sample, with the exception of Conscientiousness (Table 1).

**Table 1:** Cronbach α reliabilities for all variables encompassed in the study, for the whole sample, and separately for the clinical and non-clinical group

Scale	Whole sample	Clinical group	Non-clinical group
Autodestructiveness scale and subscales:			
Autodestructiveness (total score)	0.94	0.96	0.90
Suicidal Depression	0.91	0.93	0.86
Anxiety	0.84	0.89	0.78
Aggressiveness	0.77	0.88	0.56
Borderline	0.77	0.80	0.62
Big-five factors:			
Extraversion	0.77	0.74	0.80
Agreeableness	0.70	0.70	0.71
Conscientiousness	0.78	0.85	0.66
Emotional Stability	0.85	0.83	0.86
Intellect	0.66	0.65	0.69

The participants from the clinical group showed significantly higher values on the Autodestructiveness scale in general, as well as on Suicidal Depression, Aggressiveness, and Borderline subscales, than the non-clinical group. There were no significant differences between the two groups in the Big-Five dimensions (Table 2).

**Table 2:** Descriptive statistics for the total sample and separately for the two groups of participants, along with *t* test results for the differences between the groups*

Scale	Range	M±σ	Groups	Range	M±σ	t	*P*
Autodestructiveness scale and subscales:							
Autodestructiveness (total score)	5-95	40.18 ± 20.5	clinical	5-95	44.73 ± 22.7	**3.131**	0.002
			non-clinical	10-80	35.38 ± 16.7		
Suicidal Depression	0-30	9.89 ± 7.4	clinical	1-30	11.97 ± 8.3	**4.020**	<0.001
			non-clinical	0-22	7.69 ± 5.6		
Anxiety	1-39	16.24 ± 7.2	clinical	1-29	16.64 ± 7.4	0.720	0.473
			non-clinical	2-39	15.86 ± 7.0		
Aggressiveness	0-28	7.01 ± 5.3	clinical	0-22	7.89 ± 5.7	**2.332**	0.021
			non-clinical	0-28	6.07 ± 4.6		
Borderline	0-19	7.03 ± 3.9	clinical	0-19	8.23 ± 4.3	**4.430**	<0.001
			non-clinical	0-13	5.76 ± 3.0		
Big-Five factors:							
Extraversion	14-50	33.76 ± 6.8	clinical	14-46	33.66 ± 6.9	-0.214	0.831
			non-clinical	18-50	33.87 ± 6.7		
Agreeableness	21-50	38.74 ± 5.4	clinical	21-50	38.85 ± 5.7	0.265	0.791
			non-clinical	27-50	38.63 ± 5.2		
Conscientiousness	11-49	34.42 ± 7.1	clinical	11-49	33.68 ± 8.2	-1.436	0.153
			non-clinical	21-48	35.21 ± 5.7		
Emotional Stability	11-47	29.41 ± 8.3	clinical	11-46	28.66 ± 8.5	-1.252	0.213
			non-clinical	12-47	30.21 ± 8.0		
Intellect	21-50	35.53 ± 5.5	clinical	21-48	35.46 ± 5.9	-0.165	0.869
			non-clinical	25-50	35.60 ± 5.1		

*Significant values are in bold.

### Relations between the Autodestructiveness scale and Big-Five dimensions

The only Big-Five dimension that significantly negatively correlated with Autodestructiveness and all of its subscales was Emotional Stability (Table 3). In other words, less emotionally stable participants in general showed more autodestructiveness, but also more anxiety and aggressiveness, more borderline symptoms and higher suicidal depression. Other two Big-Five dimensions that showed significant negative correlations with the total Autodestructiveness and all but one Autodestructiveness subscales were Extraversion and Conscientiousness. Higher Extraversion pointed to generally lower Autodestructiveness, but also lower Suicidal Depression and Anxiety, and fewer Borderline symptoms, or, looking inversely, Introversion was associated with almost every aspect of Autodestructiveness. Similarly, higher Conscientiousness pointed to generally lower Autodestructiveness, but also lower Suicidal Depression and Aggressiveness, and fewer Borderline symptoms.

**Table 3:** Pearson correlations coefficients (r) between Autodestructiveness scale and its subscales, and the International Personality Item Pool scales, for the total sample (*P* values in parentheses)*

	Suicidal depression	Anxiety	Aggressiveness	Borderline	Extraversion	Agreeableness	Conscientiousness	Emotional stability	Intellect
Autodestructiveness (total score)	**0.919** (<0.001)	**0.838** (<0.001)	**0.789** (<0.001)	**0.871** (<0.001)	**-0.264**(<0.001)	-0.042 (0.572)	**-0.286** (<0.001)	**-0.716** (<0.001)	-0.054 (0.473)
Suicidal Depression	1	**0.684** (<0.001)	**0.631** (<0.001)	**0.792** (<0.001)	**-0.286** (<0.001)	-0.095 (0.207)	**-0.286** (<0.001)	**-0.685** (<0.001)	-0.085 (0.256)
Anxiety		1	**0.475** (<0.001)	**0.596** (<0.001)	**-0.357** (<0.001)	0.056 (0.460)	-0.071 (0.345)	**-0.686** (<0.001)	-0.004 (0.962)
Aggressiveness			1	**0.702** (<0.001)	0.004 (0.957)	-0.063 (0.403)	**-0.391** (<0.001)	**-0.435** (<0.001)	-0.043 (0.567)
Borderline				1	**-0.186** (0.013)	-0.060 (0.425)	-0.294 (<0.001)	**-0.591** (<0.001)	-0.056 (0,457)
extraversion					1	**0.285** (<0.001)	-0.046 (0.545)	**0.342** (<0.001)	**0.299** (<0.001)
Agreeableness						1	**0.263** (<0.001)	-0.005 (0.994)	**0.312** (<0.001)
Conscientiousness							1	**0.178** (0.017)	**0.172** (0.021)
Emotional Stability								1	0.007 (0.924)

*Significant values are in bold.

On the other hand, Agreeableness and Intellect showed no significant correlations with the Autodestructiveness scale and its subscales.

If we consider the correlations between subscales of the Autodestructiveness scale, we can see that all the subscales were in moderate to high significant positive correlations with each other (from 0.475 to 0.919, *P* < 0.01). Big-Five dimensions showed different patterns of correlation with each other, with all the significant correlations being positive and relatively low or moderate (Table 3).

### The Big-Five dimensions as predictors of autodestructive behavior symptoms

To test the main hypotheses of the study we first performed three hierarchical multiple regression analyses with Autodestructiveness as the criterion – one for the total sample and one for each of the two groups. For the total sample we entered sex in the first step and group in the second step. The third step included the five dimensions of the Big-Five model of personality, with stepwise selection. Sex and group significantly contributed to the explanation of Autodestructiveness (Table 4). However, after controlling for sex and group, two Big-Five dimensions still substantially contributed to the explanation of Autodestructiveness. The first Big-Five dimension that accounted for additional 44% of variance of the criterion variable was Emotional Stability, while Conscientiousness also significantly contributed to explaining Autodestructiveness. This finding indicates that Emotional Stability and Conscientiousness are important personality dimensions for the explanation of Autodestructiveness for both male and female adolescents, and for both the clinical and the non-clinical group. The described model accounted for 57% of variance of Autodestructiveness.

**Table 4:** Regression analyses predicting Autodestructiveness in the total sample from five dimensions of the Big-Five personality model, after controlling for sex and group*

	Autodestructiveness	
	β (*P*)	ΔR^2^
Step 1		0.030
sex	**-0.174** (0.020)	
F_(1,177) =_	5.530, *P* = 0.020	
Step 2		0.071
group	**-0.272** (<0.001)	
F_(1,176)_ =	13.992, *P* = <0.001	
Step 3		0.441
Emotional Stability	**-0.686** (<0.001)	
F_(1,175)_ =	169.048, *P* = <0.001	
Step 4		0.025
conscientiousness	**-0.164** (0.002)	
F_(1,174)_ =	10.094, *P* = 0.002	
all variables: R^2^ = **0.568**		
F_(4,174)_ =	57.227, *P* = <0.001	

*Significant values are in bold.

The second two hierarchical multiple regressions were performed separately for the clinical and the non-clinical group. The criterion was again Autodestructiveness and again the first step included sex, and the next step included the Big-Five dimensions, with stepwise selection. The results show that the findings we observed at the level of the total sample repeated in the clinical subsample (Table 5 and Table 6). After we controlled for the effects of sex, two Big-Five dimensions still significantly contributed to the explanation of Autodestructiveness: Emotional Stability and Conscientiousness. This model accounted for 62% of variance of Autodestructiveness, somewhat more than in the total sample. In the case of non-clinical group, sex did not significantly contribute to the explanation of Autodestructiveness, and only one Big-Five dimension was the significant predictor of this criterion, namely Emotional Stability. This model accounted for 45% of variance of Autodestructiveness, somewhat lower than in the case of clinical group or the total sample.

**Table 5:** Regression analyses predicting Autodestructiveness in the clinical sample from five dimensions of the Big-Five personality model, after controlling for sex*

	Autodestructiveness	
	β (*P*)	ΔR^2^
Step 1		0.103
sex	**-0.320** (0.002)	
F_(1,90) =_	10.296, *P* = 0.002	
Step 2		0.475
Emotional Stability	**-0.727** (<0.001)	
F_(1,89)_ =	99.963, *P* = <0.001	
Step 3		0.043
conscientiousness	**-0.215** (0.002)	
F_(1,88)_ =	9.982, *P* = 0.002	
all variables: R^2^ = **0.620**		
F_(3,88)_ =	47.945, *P* = <0.001	

*Significant values are in bold.

**Table 6:** Regression analyses predicting Autodestructiveness in the non-clinical sample from five dimensions of the Big-Five personality model, after controlling for sex*

	Autodestructiveness	
	β (*P*)	ΔR^2^
Step 1		0.006
sex	-0.079 (0.469)	
F_(1,85) =_	0.530, *P* = 0.469	
Step 2		0.444
Emotional Stability	**-0.671** (<0.001)	
F_(1,84)_ =	67.749, *P* = <0.001	
all variables: R^2^ = **0.450**		
F_(2,84)_ =	34.348, *P* = <0.001	

Another variable that we investigated in relation to Big-Five model was Suicidal Depression. We performed another three hierarchical multiple regression analyses, one for the total sample and one for each of the two groups, with Suicidal Depression as the criterion. For the total sample in the first step we entered sex, in the second step we entered group, in the third step the remaining three subscales of the Autodestructiveness scale, and in the last step the five dimensions of the Big-Five model, with stepwise selection. The result (Table 7) show that after controlling for sex, group, and subscales effects, two Big-Five dimensions showed independent and significant contribution to the explanation of Suicidal Depression: Emotional Stability and Agreeableness. However, the major predictors were the three Autodestructiveness subscales, especially Borderline, which accounted for most of the Suicidal Depression variance. This model accounted for 75% of variance of Suicidal Depression.

**Table 7:** Regression analyses predicting Suicidal Depression in the total sample from three subscales of Autodestructiveness and five dimensions of the Big-Five personality model, after controlling for sex and group*

	Suicidal Depression	
	β (*P*)	ΔR^2^
Step 1		0.032
sex	**-0.180** (0.016)	
F_(1,177) =_	5.900, *P* = 0.016	
Step 2		0.109
group	**-0.336** (<0.001)	
F_(1,176)_ =	22.263, *P* = <0.001	
Step 3		0.577
anxiety	**0.313** (<0.001)	
aggressiveness	**0.136** (0.020)	
borderline	**0.467** (<0.001)	
F_(3,173)_ =	117.924, *P* = <0.001	
Step 4		0.024
Emotional Stability	**-0.225** (<0.001)	
F_(1,172)_ =	16.084, *P* = <0.001	
Step 5		0.008
agreeableness	**-0.091** (0.021)	
F_(1,171)_ =	5.395, *P* = 0.021	
all variables: R^2^ = **0.750**		
F_(7,171)_ =	73.241, *P* = <0.001	

*Significant values are in bold.

In the case of clinical and non-clinical group, in the hierarchical multiple regression analyses, in the first step we entered sex, in the second the remaining three subscales of the Autodestructiveness scale, and in the last step we entered the five dimensions of the Big-Five model of personality, again with stepwise selection (Table 8 and Table 9). In the clinical group, this model accounted for 78% of variance of Suicidal Depression, and the only Big-Five dimension significantly contributing to this explanation, after controlling for sex, Anxiety, Aggressiveness, and Borderline subscales was Agreeableness. Again, the major predictors of the Suicidal Depression were the three Autodestructiveness subscales, especially Anxiety. Results in the non-clinical group were somewhat different – sex and Aggressiveness were not significant predictors of Suicidal Depression, and the only Big-Five dimension significantly contributing to Suicidal Depression explanation, after controlling for Anxiety and Borderline subscales, was Emotional Stability. This model accounted for 67% of variance of Suicidal Depression. Once more, the major predictors of Suicidal Depression were Autodestructiveness subscales, especially Borderline.

**Table 8:** Regression analyses predicting Suicidal Depression in the clinical sample from three subscales of Autodestructiveness and five dimensions of the Big-Five personality model, after controlling for sex*

	Suicidal Depression	
	β (*P*)	ΔR^2^
Step 1		0.111
sex	**-0.333** (0.001)	
F_(1,90) =_	11.235, *P* = 0.001	
Step 2		0.649
anxiety	**0.382** (<0.001)	
aggressiveness	**0.301** (<0.001)	
borderline	**0.293** (0.002)	
F_(3,87)_ =	78.600, *P* = 0.001	
Step 3		0.019
agreeableness	**-0.141** (0.007)	
F_(1,86)_ =	7.507, *P* = 0.007	
all variables: R^2^ = **0.780**		
F_(5,86)_ =	60.850, *P* = <0.001	

*Significant values are in bold.

**Table 9:** Regression analyses predicting Suicidal Depression in the non-clinical sample from three subscales of Autodestructiveness scale and five dimensions of the Big-Five personality model, after controlling for sex*

	Suicidal Depression	
	β (*P*)	ΔR^2^
Step 1		0.011
sex	-0.106 (0.329)	
F_(1,85) =_	0.965, *P* = 0.329	
Step 2		0.605
anxiety	**0.359** (<0.001)	
aggressiveness	-0.065 (0.470)	
borderline	**0.568** (<0.001)	
F_(3,82)_ =	43.181, *P* = <0.001	
Step 3		0.079
Emotional Stability	**-0.371** (<0.001)	
F_(1,81)_ =	21.107, *P* = <0.001	
all variables: R^2^ = **0.696**		
F_(5,81)_ =	37.081, *P* = <0.001	

*Significant values are in bold.

## Discussion

The results showed that clinical group had significantly higher results than non clinical group for the Autodestructiveness in general, as well as for the most subscales of the Autodestructiveness scale. The only subscale that showed no significant difference between these two groups was the Anxiety subscale. Given that all the participants from the clinical group had some of the DSM-IV diagnoses, it was expected that they would have higher results on these measures than the non-clinical group. On the other hand, the two groups showed no differences in mean results on any of the five dimensions from the Big-Five model.

The correlations between the Big-Five dimensions and the Autodestructiveness scale showed that, Emotional Instability, Introversion, and low Conscientiousness were the key dimensions related to autodestructive behavior. Our results are comparable to the results of previous studies that basically show that high Emotional Instability and Introversion are important variables when it comes to depressive symptomatology (34,46). For example, del Barrio et al (34) found negative correlations between depression and Emotional Stability and Extraversion in a sample of 423 adolescents. Similarly, Carrasco and del Barrio (36) found negative correlations of depressive symptomatology with Emotional Stability and Extraversion, but also with the remaining three dimensions of the Big-Five personality model in children and adolescents. Furthermore, highlighting the importance of Conscientiousness, John et al (45) found that boys aged 13 to 15 years with internalizing problems, whose salient features involved anxiety, somatic complaints, and social withdrawal, scored higher on Emotional Instability and Conscientiousness than non-internalizing boys.

Using hierarchical multiple regression analyses we investigated which, if any, of the Big-Five dimensions contributed to the explanation of Autodestructiveness after controlling for the sex and group effects in the total sample, and the effect of sex in separate subsamples. In the total sample, the results showed that there were two dimensions that significantly contributed to the explanation of Autodestructiveness and these were Emotional Stability and Conscientiousness. This is an interesting finding considering that Extraversion, which correlated significantly with the Autodestructiveness scale, did not prove to be a significant predictor of this variable. Furthermore, in the case of clinical sample the same two Big-Five dimensions, namely Emotional Stability and Conscientiousness, showed significant contribution to the explanation of Autodestructiveness, but in the non-clinical sample only Emotional Stability remained a significant predictor. This points to somewhat different patterns of relationships of Big-Five dimensions and Autodestructiveness in adolescents with and without psychiatric diagnoses.

We performed yet another set of hierarchical multiple regression analyses trying to explain Suicidal Depression based on the Big-Five personality model, after controlling for sex, group, and other Autodestructiveness subscales’ effects in the total sample, and the effects of sex and other Autodestructiveness subscales in separate subsamples. Once more, in the total sample, result showed that there were two dimensions that significantly contributed to the explanation of Suicidal Depression: Emotional Stability and Agreeableness. In the case of separate samples, Emotional Stability remained as the only significant predictor of Suicidal Depression in the non-clinical sample, while Agreeableness showed to be the only significant predictor in the clinical sample. It is important to highlight that these Big-Five dimensions proved to be significant predictors of Suicidal Depression, even after controlling for the effects of other subscales of the Autodestructiveness scale, which explained a large portion of variance of the criteria variable (from 58% to 65%). Furthermore, these results again point to different patterns of relationships between the Big-Five and Suicidal Depression in adolescents with and without psychiatric diagnoses, after controlling for other relevant factors.

Some limitations of the present study have to be acknowledged. The first limitation concerns different DSM-IV diagnoses of the participants in the clinical sample, as it is well documented that autodestructive behavior is regarded as a symptom of some personality disorders, namely borderline personality disorder (47). The second limitation is that in the non-clinical group there was a clear excess of female participants (74.7%). This was due to the fact that the high schools in which the participants were recruited in general had more female students.

However, none of these limitations could fundamentally affect the main outcomes of this study. Since women scored lower on Emotional Stability (48,49), and there were no substantial differences in the pattern of relations between Emotional Stability and Autodestructiveness scales and its subscales between the clinical and non-clinical group, we can conclude that sex composition of the two groups of participants had no effect on the main finding of this study.

In summary, the present research demonstrated that the Big-Five personality model can be a valid addition to the process of evaluating autodestructive behavior symptoms in adolescent psychiatric patients and adolescents without clinical diagnoses. In the performed models of hierarchical multiple regression analyses, the Big-Five model was found to account for a significant proportion of variance in Autodestructiveness and Suicidal Depression, after controlling for effects of sex, group, and in the case of Suicidal Depression, other Autodestructiveness subscales. The proposed models explained from 57% to 75% of total variance of Autodestructiveness and Suicidal Depression. Emotional Stability was the only dimension of the Big-Five model that proved to be a significant predictor in all cases, except in the case of predicting Suicidal Depression in the clinical sample. Other dimensions that showed to be important in relations of the Big-Five and autodestructive behavior were Conscientiousness in the case of Autodestructiveness, and Agreeableness in the case of Suicidal Depression. Previous studies using multiple regression analysis showed that in general population Emotional Stability and Agreeableness accounted for up to 48.3% of the variance in suicide rates, after controlling for demographics and depression (50).

In an applied perspective, our results imply that after further research, the Big-Five personality model and the IPIP questionnaire could be used as a valid addition in describing and screening autodestructive behavior in adolescent clinical and non-clinical populations.

## 

**Table:** *Significant values are in bold.
